# Teacher Evaluation of a Self-Directed Career Guidance Intervention for South African Secondary School Learners Amidst Severe COVID-19 Restrictions

**DOI:** 10.3389/fpsyg.2022.854748

**Published:** 2022-03-28

**Authors:** Izanette van Schalkwyk, Chantel Streicher, Anthony V. Naidoo, Stephan Rabie, Michelle Jäckel-Visser, Francois van den Berg

**Affiliations:** ^1^Africa Unit for Transdisciplinary Health Research, Faculty of Health Sciences, North-West University, Potchefstroom, South Africa; ^2^Department of Psychology, Faculty of Arts and Social Sciences, Stellenbosch University, Stellenbosch, South Africa; ^3^HIV Mental Health Research Unit, Department of Psychiatry and Mental Health, Faculty of Health Sciences, University of Cape Town, Cape Town, South Africa; ^4^Department of Industrial Psychology, Faculty of Economic and Management Sciences, Stellenbosch University, Stellenbosch, South Africa

**Keywords:** career guidance, secondary school learners, teachers, low-income communities, resource-constrained schools, mixed method

## Abstract

The South African government’s COVID-19 pandemic risk mitigation strategies significantly limited social contact, which necessitated a novel approach to existing face-to-face career guidance practices. The Grade 9 Career Guidance Project, originally developed as a group-based career development intervention, required radical adaptation into a self-directed, manualized format to offer career guidance to Grade 9 learners from low-income communities amid a global pandemic. The adaptation and continuation of the project was deemed essential as secondary school learners in low-income communities have limited career guidance support. Furthermore, a close collaboration with the teachers at eight resource-constrained South African secondary schools was vital for successful implementation. To assess the success of the adaptation to a self-directed format, a mixed-methods design was employed, and Life Orientation teachers’ evaluative feedback was solicited (*n* = 11). Favorable quantitative results were obtained; majority of teachers agreed that learners enjoyed the booklet (manualized format) and that it was deemed an adequate substitute to the previous contact-based format of the Career Guidance Project. This was also confirmed by the qualitative findings revealing teachers’ satisfaction with the booklet’s content, specifically that the booklet is complementary to the Life Orientation curriculum. Qualitative findings identified specific contextual barriers that contributed to some learners struggling to use the booklet optimally. The results suggest that it is feasible and acceptable to implement a self-directed career guidance intervention among secondary school learners amid a global pandemic. Teachers recommended ways to integrate the booklet, resources, and contact sessions as a preferred way forward. These findings have important implications for similar resource-constrained settings that may not have readily access to in-person career guidance and counseling human development.

## Introduction

Since the outbreak of the COVID-19 pandemic, the adverse effects of the pandemic to people’s physical health and mental well-being (Wong, 2020), the world economy and employment (Chen and Fellenz, 2020; Sánchez-Páramo, 2020; Bux, 2021) are undeniable. The increase in prevalence of mental health problems has become apparent. However, Wong (2020) cautions that prior to COVID-19, the world was already facing a mental health crisis. For example, nearly 800,000 people die by suicide every year [World Health Organization (WHO), 2021] and suicide is the fourth leading cause of death among the 15–19-year age cohort globally (77% of global suicides occur in low- and middle-income countries). Unmistakably, in the era of COVID-19, the complexities of being well and positive functioning (cf. PP 3.0) are emphasized, since people are bombarded daily by news of the rising death toll, new cases, and unemployment numbers, whilst in social isolation (Yıldırım et al., 2021). Yet, it is important to note that macro-systemic risk factors such as global economic and systemic discrimination have been evident prior to the onset of the pandemic. Particular vulnerable groups, such as adolescents in low-income communities, are dealing with the adverse impact of the pandemic layered on extant multiple individual, community, and environmental stressors (Sánchez-Páramo, 2020). Problems in living–such as quality of life and livelihood for adolescents growing-up in challenging contexts–are intensified in times like the COVID-19 pandemic.

One of these prevailing contexts is the limited access to adequate career guidance for many South African secondary school learners, specifically learners living in low-income communities (Jäckel-Visser et al., 2021). Jonck and Swanepoel (2019) describe career guidance as those services and activities aimed at assisting individuals at any point in their lifespan “…to make educational, training and career decisions as a means to ultimately manage their chosen profession” (p. 2). One of the first career milestones for the South African secondary school learner is formally selecting the subjects in Grade 9 for the final phase of their secondary education (Grades 10–12). The role and provision of career guidance and counseling at secondary school level are critical in supporting young adolescents in taking initial steps in formulating post-school study and career goals (Hartung et al., 2005; Jäckel-Visser et al., 2021). In low-income settings, external factors such as economic deprivation, high youth unemployment rates, lack of access to career services and resources and absence of parental support severely undermine the career development process (Dube, 2019). Many of these external factors can be viewed as rooted in the historical and socio-political impediments created through apartheid in South Africa (Naidoo et al., 2017), which altered the inclusive nature of successful transitioning from school to the vocational environment (Spaull, 2012; Jonck and Swanepoel, 2019). On top of these challenges associated with “inherited poverty” (Burger et al., 2014; van Schalkwyk, 2020), Jonck and Swanepoel (2019) found that school type (indicated as historically advantaged and disadvantaged schools) has a statistically significantly influence on career guidance service delivery and career path knowledge gained from career guidance. This is the reality for many South African secondary school learners, who have to contend with limited support and access to resources in the process of making important career-related decisions (Rabie et al., 2021).

The impact of the highly infectious global COVID-19 pandemic in 2020 [World Health Organization (WHO), 2020] not only affected people’s health, but also changed the world of work profoundly (Maree, 2021). Numerous emergency actions, such as strict lockdown measures, were implemented by the South African government to curb the spread of the disease (Ramaphosa, 2020). This announcement, however, had a direct impact on all South African secondary schools resulting in sudden extended school closures, a hasty adaption to remote learning modalities as well as enforcing a class rotation model, where half of each class attends school every alternate week. These measures also implied limited teacher-to-learner contact time and less time to cover the normal curriculum via remote learning modalities (Ramrathan, 2021). A further consequence was the direct impact on traditional career guidance and counseling offered at schools as part of the Life Orientation curriculum. Pillay (2021, p. 5) describes the subject Life Orientation as the “bridge to career guidance” that should not be underestimated given its importance in helping learners with possible career paths and development opportunities.

Given the evolving social restrictions, the established *modus operandi* of the Career Guidance Project (CGP) was adapted from an “in-person,” group format, career counseling intervention to a self-directed career guidance intervention aimed at assisting Grade 9 secondary school learners in making informed career decisions (Jäckel-Visser et al., 2021). In previous years, the in-person intervention comprised psychometric testing followed by interactive workshops [See Naidoo et al. (2019) and Rabie et al. (2021) for a more detailed description]. The reformulation of the project entailed the development of the Self-directed Career Guidance Booklet [Self-directed Career Guidance Booklet (SCGB), 2020] for the Grade 9 learners to complete individually complemented by electronically-based supplementary resources (video content, an interactive website with career and study-related resources). In the process of adapting the project it became vital to incorporate Life Orientation (LO) teachers’ input, given that these teachers play an instrumental part in reinforcing learners’ self-efficacy, which is not only relevant for their academic success (van Deventer, 2009), but also for the planning of their career paths (Jonck and Swanepoel, 2019). Thus, a mixed-methods evaluative design was used to obtain feedback from the LO teachers from the eight secondary schools involved in this project to ascertain the feasibility and acceptability of the self-directed career guidance project from the LO teachers’ perspectives. The main aim of this paper is to evaluate the teachers’ perceptions of the implementation of a self-directed career guidance intervention for South African secondary school learners at 8 schools amidst severe COVID-19 restrictions.

According to the Cordaid Annual Report (2015), adolescents represent the upcoming workforce of society, investing time and effort into this demographic can result in a massive boost for the economy. Given that the adolescent population is significantly large in Sub-Saharan Africa where half of the youth population is under the age of 18 (Philipps, 2018), this point of departure has implications for their positive functioning (or not) and sustainable development.

## Conceptual and Theoretical Background

In this section we discuss briefly the conceptual cornerstones associated with Community Psychology and the theoretical framework of Positive Psychology.

### Positive Psychology

Positive Psychology (PP) has emerged as an esteemed science of well-being where the magnificence and complexities of healthy functioning and meaningful living could be explored and studied (Linley and Joseph, 2004, p. 4). Already in 1961, Szasz (1961 in Wong, 2020) pointed out that “human difficulties should be conceptualized as problems in living rather than mental illnesses or diseases” (p. 568). In other words, struggling with life’s problems cannot be understood merely as the eradication of “the bad and the ugly,” but also entails the study and deliberate efforts to protect and promote personal, relational and collective well-being [cf. Seligman (2011)]. In alignment with being well and living well (Lomas, 2016), this initial embrace of the positive was referred to as the “first wave” of PP. This focus of Positive Psychology can enrich approaches to career development (Robertson, 2017) when considering the wide range of different career development theories mapping career development and career choice (Coetzee and Schreuder, 2021). For this intervention a post-modern career counseling approach was considered. Maree (2018) has advocated for career counseling to shift from an objective approach to an interpretative process using new and creative ways of assessment. Career counseling should not be a linear process with the counselor merely administering and scoring an interest inventory and then providing recommendations to the client based on these scores (Morgan et al., 2019). Instead, career counseling should be a recursive process with both the counselor and client actively engaged in constructing meaning (McIlveen and Patton, 2007; Maree, 2018) in factoring in the realities of the client’s family and community contexts (Albien and Naidoo, 2017). In such an approach, career counselors become facilitators rather than prescriptive experts, and clients are able to speak, act, think, and choose for themselves. Clients are encouraged to construct their own meaning in the career exploration process, and to take responsibility for their own choices and development. Added to Maree’s (2018) advocacy for post-modern career counseling practice to reflect innovative methods, techniques and structures, is the utilization of group-based career guidance as a methodology. Naidoo et al. (2019) aver that there is increasing evidence, although not always explicitly stated, that group-based interventions may be effective in achieving career behavioral outcomes in the school context and in reinforcing a strengths approach, particularly in disadvantaged communities [See Rabie et al. (2021)].

The original premise of PP with its focus on the positive was challenged (Lomas and Ivtzan, 2016; Lomas et al., 2020) and PP was frequently criticized for placing the “responsibility of well-being and success entirely on individuals without considering systemic or structural biases against disadvantaged groups” (Mead et al., 2019; Wong, 2020, p. 275). For example, Wong (2020) states that regrettably research focused mostly on the “what and how” of happiness as the pleasant life (hedonic well-being) and the flourishing life, i.e., functioning well (eudaimonic well-being) (Delle Fave et al., 2016; Wong, 2020) with little attention given to relevant contextual factors, and prolonged suffering. Therefore, complementing PP’s focus on “the neutral and positive territories of life, Second Wave Positive Psychology (PP 2.0) recognized that, for most people, life is lived in negative territories” (Wong, 2020, p. 276). Well-being is re-conceptualized as “both the process and the outcome of managing an adaptive balance between pursuing positive goals and overcoming or transforming all the negative forces” (Wong, 2020, p. 568). The transcendence of trials and tribulations - integral to sustaining well-being–aligns with resilience studies and is evident in the narratives of South African youth in overcoming adversity (Theron, 2017).

Due to the self-directive and progressive tendency of science, PP 2.0 opened up new avenues of research and applications while avoiding many of the problems inherent in the initial wave of PP (Wong and Roy, 2018). The second wave (PP 2.0) is concerned with how to bring out the best in individuals and society in spite of and because of the dark side of human existence through the dialectical principles of yin and yang. This distinct shift from focusing merely on individual happiness and success to individual well-being and the bigger picture of humanity can be explained in the following example: when a secondary school learner living in a low-income community fortifies his/her personal [character] strengths, such as courage (e.g., being a person of integrity) and wisdom (e.g., making plans to study well), then he/she could become a more resilient human being and make this world a better place. Furthermore, PP 2.0 pivots around the universal human capacity for meaning seeking and meaning making (Wong, 2015) in achieving optimal human functioning under both positive/enabling and challenging conditions. This was aptly described by Wong (2020) as “… living a better life requires efforts to improve both one’s own life and the world we all live in” (p. 569). Consequently, PP 2.0 is better equipped to address issues associated with existential suffering during COVID-19 (Wong, 2019, 2020).

#### Positive Psychology and 3rd Wave (PP 3.0)

Currently there are indications of PP in a third wave with a more “explicit focus on contextualization, inter-connectedness (including spirituality/transcendence) as well as multi-inter-and transdisciplinarity” (Wissing et al., 2020, p. 17). This entails a more comprehensive understanding of well-being [cf. definition of Wong (2020)] which embraces both positivity and negativity [see Lomas et al. (2020)]. The need to include the role and impact of context, social justice and values also belongs to the PP 3.0 as argued by Di Martino et al. (2018), as well as interconnectedness among various levels and domains of human functioning (Wissing et al., 2020). Finally, PP 3.0 multi-, inter-, and transdisciplinary approaches can add to the disentanglement (versus the un-doing) of well-being and its complexities (van Schalkwyk and Naidoo, 2021; Wissing, 2022).

### Community-Based Participatory Research

Community Psychology as a discipline recognizes that mental health and well-being transcends the individual, and therefore the discipline rather focuses on situating individuals within multiple social systems and environmental contexts to holistically conceptualize well-being (Riemer et al., 2020). Thus, community psychology from a social justice imperative identifies community engagement initiatives as a vital tool through which to enable social change and make a tangible difference in people’s lives (Riemer et al., 2020). The CGP identified the inequality of access to adequate career guidance services within the South African education system (specifically within resource constrained schools) as a social justice issue to redress (Naidoo et al., 2019). Thus, the CGP, as a community engagement initiative, aimed to equip Grade 9 learners with the knowledge and resources to make informed subject choices and career plans to thereby, support the learners’ career development (Rabie et al., 2021). As key partners, the input of the LO teachers is crucial to the design, delivery, and adaptation of the CGP.

Community engagement initiatives are, however, not infallible; researchers have critically examined the practices of community engagement specifically when it comes to the power imbalances between academics and the communities wherein they intervene (Ogunniyi, 2011). The Community-based Participatory Research (CBPR) approach emanated from this critical community psychology lens (Lazarus et al., 2015). Community initiatives are guided by the intention of understanding and respecting the historical context of a community, and the current relational dynamics, to ensure that the academic objectives of an initiative is aligned with the needs of the community and, to ultimately promote the longevity and sustainability of an initiative (Lazarus et al., 2012, 2015). The CBPR approach recognizes that community members’ lived-experiences and knowledge of their community are pertinent to the sustainability of any intervention. Thus, the CBPR approach necessitates the inclusion of community members in the planning, implementation, and assessment of a community intervention to promote community ownership and empowerment (Lazarus et al., 2015; Churchman et al., 2017). The formation of community partnerships enables community engagement initiatives to become long-term interventions (Lazarus et al., 2012), and thus these partnerships need to be nurtured and built upon to remain sustainable. Hence teacher feedback was a crucial part of the iterative loop in assessing this adapted career guidance intervention.

## Research Design

A mixed methods research design was utilized in this study, as the combination of structured and explorative data collection approaches allowed more latitude to ascertain the LO teachers’ unique perspectives of the self-directed CGP (Bryman, 2012; Clark and Ivankova, 2017). The mixed methods survey enabled the project team to gain the perspectives of a larger sample group of teachers involved in the project as they could complete the surveys in their own time electronically. Moreover, the open-ended questions provided the teachers space to include additional comments and reflections on the adapted version of the project that were not addressed in the quantitative portion of the surveys.

### Sample

The participant population consisted of 17 LO teachers from the eight secondary schools involved in the CGP. Eligibility for the study required that the participant be currently teaching Grade 9 learners (from one of the eight secondary schools involved in the CGP) and provided informed consent. Through a convenience sampling approach (Bryman, 2012), a subsample (*n* = 11) of LO teachers provided feedback on the CGP.

### Setting

Extant research indicates that South African resource constrained schools face significant barriers in providing historically underserved learners with adequate career guidance services (Pillay et al., 2014; Albien and Naidoo, 2017; Modiba and Sefotho, 2019). In particular, research findings indicate that LO teachers are not effectively trained in administering career guidance and face significant time constraints when it comes to addressing all the components of the LO curriculum (Maree, 2013; Smit et al., 2015). Thus, when it comes to career development, learners in many low-resourced schools are not being properly supported (Pillay, 2021). The CGP was initiated with the social justice imperative of providing career guidance and support to Grade 9 learners in resource constrained communities with their career planning and subject choice (Naidoo et al., 2019; Rabie et al., 2021). The contact-based format of the CGP had to be fundamentally adapted in response to the severe COVID-19 restrictions instated by the South African government. The new self-directed format and implementation of the CGP could not be piloted in the short implementation timeframe. Thus, the quantitative and qualitative feedback collected from the LO teachers regarding the booklet and supplementary resources were integral in the evaluation of the adapted CGP. Learner feedback on the CGP has been reported elsewhere (Jäckel-Visser et al., 2021; Streicher, 2021).

### Data Collection Measures

Data were collected through a study-constructed survey. The survey comprised of 12 items measured in a five-point Likert scale, ranging from strongly disagree (1) to strongly agree (5). In addition, the survey included a qualitative item. The quantitative items of the survey assessed three components: Booklet Acceptability, Supplementary Resources Acceptability, and Reflection on New Format, whereas the qualitative component measured Future Recommendations for the improvement of the intervention. These items were developed by the project team after the implementation of the CGP specifically for the purposes of this study; intended to assess the necessary changes made to the original format and implementation of the CGP amid a global pandemic.

#### Booklet Acceptability

Six statements surveyed the acceptability of the self-directed career guidance booklet. Respondents were asked to rate six items on a 5-point Likert Scale (from strongly disagree to strongly agree). For instance, “From your perspective as a Life Orientation teacher, the information in the self-directed career guidance booklet helped the Grade 9 learners, with their subject choices.” The six items were collated into a total score ranging from 6 to 30, with lower scores indicating a negative evaluation and higher scores a positive evaluation. In addition, LO teachers were asked five open-ended questions pertaining to their evaluation of the acceptability of the booklet, for example, “Describe how you used or will use the booklet during LO class time.”

#### Supplementary Resources Acceptability

Four statements evaluating the acceptability of the supplementary resources used in the project were rated on a similar 5-point Likert Scale (from strongly disagree to strongly agree). For example, “The website that was developed for the project is a helpful resource for the Grade 9 learners.” The four items were aggregated into a total score (ranging from 4 to 20), with lower scores indicating a negative evaluation and higher scores a positive evaluation of the supplementary resources.

#### Reflection on New Format

Two statements, evaluating the self-directed format of the CGP, were assessed on a Likert Scale ranging from strongly disagree to strongly agree. The two items were computed into a total score (ranging between 2 and 10), with lower scores indicating a negative evaluation and higher scores a positive evaluation of the self-directed format of the CGP.

#### Future Recommendations

An open-ended question was used to ask LO teachers to identify how the CGP can be improved: “How can the self-directed career guidance booklet be improved for future projects?”

The survey was e-mailed to all 17 LO teachers from the eight secondary schools.

### Ethical Measures

The Stellenbosch University Research Ethics Committee provided approval for the implementation of the CGP (Project 3072). In addition to the Western Cape Education Department granting institutional approval (20180301-9937) for the study, each of the eight schools’ principals also granted permission. Informed consent was also obtained from all participants. Each participant was made explicitly aware that their participation was voluntary, and that they could withdraw at any point without any negative implications. In addition, the participants were assured that the confidentiality of their identity and responses would be respected and protected.

### Data Analysis

The quantitative data collected were analyzed using IBM SPSS Statistics (Version 24). The quantitative test variables derived from the LO teachers survey data were interpreted in accordance with this study’s quantitative objectives. The reliability of each scale was determined using Cronbach’s alpha coefficient, with obtained scores exceeding.70 indicating sufficient reliability. The qualitative data collected from the LO teachers’ survey were analyzed using Atlas TI.9 using a thematic analysis approach (Bryman, 2012). The thematic analysis entailed the generation of codes applied to the sentences, words or phrases of the participants’ responses that symbolized a theme present in the data. Once a codebook was generated, the codes were re-examined and refined to ensure they accurately represented the themes present in the data. The qualitative data were triangulated among three coders, blinded to the other’s initial codes, to support the consistency and trustworthiness of the qualitative data analysis (Bryman, 2012).

## Results

### Life Orientation Teacher Survey Quantitative Results

#### Booklet Acceptability

The Booklet Acceptability subscale demonstrated sufficient reliability, with α = 0.796. The scores presented in Table 1 below indicate that majority of the LO teachers (*n* = 11) positively endorsed the self-directed career guidance booklet (*M* = 26.18; SD = 2.750). The mode scores of each individual question were interpreted to identify the most frequently endorsed response. Questions 1–5, excluding Question 3 received a mode score of 5, which indicated that the majority of the LO teachers strongly agreed that the booklet helped the Grade 9 learners with their subject choices (Question 1) and career goals and plans (Question 2). In addition, majority of the LO teachers strongly agreed that the booklet instructions were clear and easy to follow (Question 4) and that the questions were age appropriate (Question 5). Furthermore, Questions 3 and 6 received a mode score of 4, indicating that most of the LO teachers agreed the booklet was an adequate replacement to the previous contact-based format of the CGP (Question 3) and that they perceived that the learners enjoyed the booklet (Question 6).

**TABLE 1:** Descriptive statistics of life orientation teachers’ perceptions of the booklet acceptability.

Variable	*n*	*M*	SD	Median	Minimum	Maximum
Booklet acceptability	11	26.18	2.750	27	22	30

The Supplementary Resources Acceptability subscale demonstrated sufficient reliability, with α = 0.838. Table 2 presents the descriptive statistics of the Supplementary Resource Acceptability scores among the LO teachers (*n* = 11). The Supplementary Resources Acceptability component attained a mean score of 13.909 out of 20 (SD = 3.989). The mean average score indicates that the supplementary resources were evaluated as neutral. Upon examination of the mode values of the individual questions, Questions 9 and 10 that assessed the electronic and physical resource kits developed for the CGP website attained a mode score of 4, which indicates that majority of the LO teachers rated these resources to be helpful to the Grade 9 learners. Question 7 attained a mode score of 3, which indicates that most of the LO teachers were ambivalent to whether the video content produced was helpful to the Grade 9 learners. Question 8 received bimodal scores of 3 and 4, which indicate that the LO teachers were slightly in agreement that the video content was an adequate replacement to the contact-based workshops implemented in the past. In sum, the variance in the evaluation of the supplementary resources indicated marginal acceptability.

**TABLE 2:** Descriptive statistics of life orientation teachers’ perceptions of the supplementary resources acceptability.

Variable	*n*	*M*	SD	Median	Minimum	Maximum
Supplementary resources acceptability	11	13.909	3.986	15	4	20

#### Reflection on New Format

This component was found to be reliable, with α = 0.738, and attained a mean score of (*M* = 3.09) out of 5 (SD = 1.044). Thus a majority of the LO teachers indicated slight preference for the self-directed format and implementation of the CGP.

#### Life Orientation Teacher Survey Qualitative Results

The thematic analysis of the LO teachers’ open-ended response feedback on the LO teacher survey produced four prevalent themes. The themes *Booklet Content Acceptability, Supplementary to Curriculum, Contextual Barriers to Completion* and *Recommendations for the Future* will be discussed in the section to follow.

#### Booklet Content Acceptability

The LO teachers positively endorsed the content of the self-directed career guidance booklet. Some expressed that the booklet itself was “very detailed,” “comprehensive,” and that “no improvements to the content is necessary.” In addition, the LO teachers complemented the layout and presentation of the booklet as “very colorful and fun.” One respondent expressed that the language in the booklet was “age-appropriate.” Overall, the booklet was credited as “learner-friendly” and that it “caters for all types of learners.”

The self-directed career guidance booklet [Self-directed Career Guidance Booklet (SCGB), 2020] sought to provide Grade 9 learners career guidance and subject choice support. The LO teachers credited the booklet content as “crucial for career choices” and indicated that the booklet helped the Grade 9 learners to make “clear subject choices,” a formal expectation for Grade 9 level.

#### Supplementary to Curriculum

The LO teachers expressed that the booklet content supported the LO career guidance and subject choice curriculum requirements; one respondent indicated “the booklet speaks to the curriculum.” Several LO teachers indicated that the booklet is a useful resource to use in LO lessons. The LO teachers expressed that they would be able to use the booklet as a resource when addressing “career subject,” “career guidance,” and the learners’ school-based LO assessment task.

#### Contextual Barriers to Completion

The LO teachers identified several contextual challenges the Grade 9 learners faced as a barrier to their completion of the booklet. A lack of parental guidance and support, or “absent parents” was identified as a hindrance to booklet completion as “learners do not have proper support at home to help and guide them through the booklet.” Moreover, the COVID-19 pandemic and associated challenges were identified as a covert barrier. For instance, poor learner attendance at school led to some learners not receiving the booklet. Moreover, a local bus strike in one community further affected learner attendance, as some learners lived beyond walking distance to the school. In addition, the COVID-19 pandemic imposed time constraints to the LO curriculum, curtailed in-person lesson time due to each school’s varying COVID-19 schedule meant that the LO teachers “did not have enough time to guide them (the learners) through it (the booklet) page by page.” Financial constraints at home may also limit learners from accessing online resources.

#### Insights for Future Iterations of Project

In addition, the LO teachers provided helpful insights and recommendations for future iterations of the project. Firstly, the LO teachers suggested a hybrid implementation of the CGP as an “integration of the booklet, resources, and contact sessions” in future. Secondly, to redress the WIFI and data constraints (due to costs) the schools and learners faced, a LO teacher recommended that Grade 9 learners should be allowed access to computer labs. Thirdly, to improve the booklet’s complementary nature to the LO curriculum, a LO teacher suggested integrating the LO curriculum assessment tasks into the booklet. Lastly, an LO teacher proposed implementing a career fair at the schools to provide the Grade 9 learners more practically with career and study-related information.

## Discussion

Results of this mixed method research to ascertain LO teachers’ unique perspectives of the feasibility of the self-directed CGP yielded favorable and reliable quantitative results supported by qualitative findings. While the quantitative results clearly indicate that teachers felt that learners enjoyed the booklet as an adequate substitute to the previous contact-based iterations of the CGP, the qualitative findings offer a more nuanced understanding of the teachers’ approval of the booklet’s content. The strongest endorsement was that the teachers regarded the booklet as complementary to the Life Orientation curriculum in the South African secondary school system. The blending or integration of these findings provides a stronger understanding of the research question [see Creswell (2014), p. 215] by revealing the impact of context and its limiting (and possibly) long-term implications for the CGP. Next, we present a brief discussion of the findings and its implications in the broad South African context in order to offer directions for future research.

The quantitative results indicated that LO teachers positively evaluated the self-directed career guidance booklet as the primary resource of the CGP and that it interfaced well with the LO curriculum. The majority of the LO teachers strongly agreed that the booklet helped the Grade 9 learners with their subject choices. The teachers concurred that the instructions of the booklet were clear and easy-to-follow, and, the content and the presentation of the information in the booklet was age-appropriate. These appraisals were further substantiated by LO teachers expressing their appreciation for the electronic and physical resource kits and the CGP website. Teachers appeared more tentative about the interface between the booklet and the supplementary resources. Nevertheless, these findings are important given that Prinsloo (2007) found that most LO teachers were not optimistic about the long-term effect of LO teaching and they were concerned about the insufficiency of their training to teach LO (Rooth, 2005). Providing accessible resources can assist LO teachers achieve their career guidance curriculum mandate.

It is significant that LO teachers regarded the self-directed career-guidance booklet as a helpful resource in teaching and guiding learners about subject- and careers choices. The role of teachers in implementing this intervention amidst severe COVID-19 restrictions in 2020 was of key importance, since challenging problems in the education system in a highly unequal South African context add to existing poverty spirals (Spaull, 2013). The continued provision of unequal quality education (a legacy of apartheid education) remains a poverty trap mechanism (Burger et al., 2014). The South African education system has among the highest variations in education outcomes: schools from the bottom income quintile markedly underperform relative to schools in the top quintile (Branson and Zuze, 2012; Burger et al., 2014). The role of teachers and their training in providing career guidance is considered one of the determining factors in orientating youth for the labor market (Spaull, 2013). The need for sound career guidance with appropriate resources at secondary school level is vital; Makola et al. (2021) emphasize that Life Orientation can play a seminal role to prepare learners for the career exploration and planning process. Moreover, especially with the COVID-19 restrictions and potential school closures, LO teachers may benefit from having access to a range of resources to implement their career guidance activities online with their learners.

The LO teachers provided valuable evaluative feedback on the adaptation of the CGP from a face-to-face, group based format to a self-directed format in which the individual learner can work through a set of self-exploration activities to gauge their self-knowledge and career related interests. The qualitative feedback, however, needs to be disaggregated to be of value to identify the pertinent recommendations to improve the implementation of the CGP. Firstly, there was unanimous approval of the self-directed approach (with its colorful content and structured activities format) as addressing a cogent learner need and a seminal LO curriculum objective under difficult circumstances. The learners were provided with a set of resources that assisted them to (1) explore their career interests; (2) complete the prescribed LO assignment task, and (3) had a structured basis to assist them with choosing their school subjects for the final phase of high school. Secondly, the teacher feedback specifically endorsed the utility of the self-directed booklet. The self-directed intervention promote a sense of learner agency and empowerment (Morris, 2019) requiring the individual learner to take responsibility for their own learning and development by working through a manualized booklet (Streicher, 2021). The findings of several research studies have indicated that self-directed learning can positively enhance learners’ career decision-making abilities (De Bruin and Cornelius, 2014; Briska and Dislere, 2018). Teacher feedback endorsed that the self-directed career guidance booklet [Self-directed Career Guidance Booklet (SCGB), 2020] played a facilitative role in assisting Grade 9 learners engage with linking their career related interests, knowledge of self, with their subject choices. The LO teachers credited the booklet as being user-friendly and age-appropriate with its content as inspiring learners to be reflective and informed about their career decisions and their subject choices, a formal expectation for Grade 9 level. With schools shut down for several months during the COVID-19 pandemic period, these resources provided learners with valuable information, guidance and support, moreover, in a self–reflective format that they could follow at their own individual pace. This was of particular relevance for the teachers who expressed that the booklet content strongly supported the LO career guidance and subject choice curriculum requirements, and could be as a resource in LO lessons and to learners with their school-based LO assessment task.

The LO teachers, however, identified several contextual challenges that needed to be addressed. The concern about “absent parents” and the related need for parental guidance and support for the learners with their career aspirations were identified as important considerations, especially in low-resourced communities. Albien and Naidoo (2018) have previously identified the inclusion of parents as being a crucial enabler in this process of factoring in family, culture and socio-economic realities that may impact learners’ choices. Here, information about accessing government bursaries (contained in the booklet) for example, may help provide options that parents can consider. Sessions with parents supplemented by an information booklet for parents have been recommended in helping to establish a recursive career guidance process. The LO teachers also recommended using a hybrid implementation of the CGP with the implementation of the booklet and resources being augmented by facilitated workshop sessions. Learner feedback [see Jäckel-Visser et al. (2021)] also advocated for the need for direct online interaction especially during the Covid-19 restriction conditions. Making zero-rated (free or sponsored) WIFI facilities available may assist learners to access online resources when they do not have access to the school’s computer labs. From the insights gained from the findings, we recommended that future research explores how the self-directed career guidance booklet can be effectively implemented and used as part of the Grade 9 Life Orientation curriculum. A resource guide can be developed to assist LO teachers with this task.

A clear limitation of this study is its small, purposive sample. All participants were recruited from 8 partner schools in low-income communities, using convenience sampling with universal inclusion. Therefore, we cannot exclude the possibility that the sample may be underrepresented and positively biased, hence potentially impacting the findings and limiting the generalizability of the results. COVID-19 restrictions extant in the schools at the time of data collection meant that uninform data collection methods could not be applied, hence the decision to revert to emailed surveys.

## Conclusion

The findings of this study confirm South African Life Orientation teachers’ support for the use of a self-directed manualized booklet to assist Grade 9 learners with exploring their career interests, reflecting on their knowledge of self, and linking these aspects to choosing their school subjects for the final phase of secondary school. There was consensus that the self-directed booklet was a useful career guidance resource that learners could use on their own (as during COVID-19 related school closures). Teachers indicated that as the booklet had strong overlaps with the Grade 9 LO curriculum, they could utilize it as part of their lesson plans. The teachers appeared more tentative about the interface between the booklet and other supplementary resources (electronic resources and the website) which may require the use of technology and data. Financial constraints may impede the access to these electronic resources for learners in low-income communities. Hence, a self-directed career guidance booklet may serve as an important resource for learners in low-income contexts where financial constraints may limit their access to internet based resources. Teachers, however, strongly recommend that the booklet be augmented with face-to-face, namely ‘in person’ discussions in the classroom.

## Data Availability Statement

The original contributions presented in the study are included in the article/supplementary material, further inquiries can be directed to the corresponding author.

## Ethics Statement

The Stellenbosch University Research Ethics Committee provided approval for the implementation of the CGP (Project 3072). In addition to the Western Cape Education Department granting institutional approval (20180301-9937). The patients/participants provided their written informed consent to participate in this study.

## Author Contributions

All authors listed have made a substantial, direct and intellectual contribution to the work, and approved it for publication.

## Conflict of Interest

The authors declare that the research was conducted in the absence of any commercial or financial relationships that could be construed as a potential conflict of interest.

## Publisher’s Note

All claims expressed in this article are solely those of the authors and do not necessarily represent those of their affiliated organizations, or those of the publisher, the editors and the reviewers. Any product that may be evaluated in this article, or claim that may be made by its manufacturer, is not guaranteed or endorsed by the publisher.
